# Estimation of β-ray dose in air and soil from Fukushima Daiichi Power Plant accident

**DOI:** 10.1093/jrr/rrt209

**Published:** 2014-02-05

**Authors:** Satoru Endo, Kenichi Tanaka, Tsuyoshi Kajimoto, Nguyen Tat Thanh, Joji M. Otaki, Tetsuji Imanaka

**Affiliations:** 1 Quantum Energy Applications, Graduate School of Engineering, Hiroshima University, 1-4-1 Kagamiyama, Higashi-Hiroshima 739-8527, Japan; 2 Center of Medical Education, Sapporo Medical University, S1W17, Chuo-ku, Sapporo 060-8556, Japan; 3 The BCPH Unit of Molecular Physiology, Department of Chemistry, Biology and Marine Science, Faculty of Science, University of the Ryukyus, Nishihara, Okinawa 903-0213, Japan; 4 Research Reactor Institute, Kyoto University, 2, Asashiro-Nishi, Kumatori-cho, Sennan-gun, Osaka 590-0494, Japan

**Keywords:** Fukushima Daiichi Nuclear Power Plant accident, β-ray dose, radiotellurium, radioiodine, radiocesium

## Abstract

Following the Fukushima Daiichi Nuclear Power Plant (FDNPP) accident of 2011, which deposited radionuclides across Tohoku and northern Kanto, β-ray dose evaluation has been performed to estimate radiation exposure for small creatures like insects as well as human skin. Using the Monte Carlo radiation transport code MCNP-4C, we calculated the β-ray dose for ^129m^Te, ^129^Te, ^131^I, ^132^Te, ^132^I, ^134^Cs and ^137^Cs in air as a function of altitude and in soil. These calculations of β-dose rate for each radionuclide were conducted for the conditions following the FDNPP accident, with ^137^Cs deposition assumed to be 1000 kBq/m^2^. Beta-ray dose rate was found to be ∼10-fold (resp. 5-fold) higher than the γ-ray dose rate in the soil (resp. on the ground surface) at ∼20 days after deposition, and ∼4-fold (resp. 1.7-fold) higher after 6 months or more. For convenience, the height dependence of the ratio for 0, 10, 30, 90, 180 and 365 days after deposition was obtained by a fitting function. The cumulative 70 µm β-ray dose at 30, 60 and 90 days after deposition was estimated to be 35, 45 and 53 mGy for the ground surface, and 61, 79 and 92 mGy in the soil, respectively. These results can be used to estimate the external β-ray exposure for small creatures as well as for human skin.

## INTRODUCTION

The nuclear accident at the Fukushima Daiichi Nuclear Power Plant (FDNPP) occurred as a consequence of the massive earthquake and associated tsunami that struck Japan on 11 March 2011. The radionuclides that were released as a result were deposited over a wide area from the Tohoku region to the northern Kanto region [1]. Because β-rays are not directly related to effective dose, dose evaluations have primarily focused on γ-rays. Beta-rays are easily shielded by the surfaces of tissue of even a few millimeters thickness, and are therefore usually discussed using the 70 µm dose equivalent in the context of skin dose [2, 3].

Hiyama *et al.* reported that the first-voltine adults of the pale grass blue butterfly (*Zizeeria maha*) collected in the Fukushima area in May 2011 showed relatively mild abnormalities [4]. More severe abnormalities were observed in the F1 offspring from the first-voltine females, which were then inherited by the F2 generation. They concluded that artificial radionuclides from the FDNPP accident caused physiological and genetic damage to this species.

The eggs and larvae of the pale grass blue are 1–2 mm and oblate, or 3–25 mm and prolate; therefore, β-rays effectively transfer their energy through the whole volume of the organism. Since larvae of the pale grass blue both crawl on the ground on warm days and also burrow into the soil on cold days, they are exposed to β-rays from radioactive cesium, iodine and tellurium isotopes deposited on the ground. There is therefore a possibility that their reported genetic and physiological abnormalities might have been caused by radiation, such as γ- and β-rays from the radionuclides released from the FDNPP. To estimate the radiation exposure to small creatures and human skin, both the β-ray dose in the air as a function of altitude, and the β-ray dose in the soil are reported here.

## MATERIALS AND METHODS

### Monte Carlo transport calculation

**Fig. 1.**
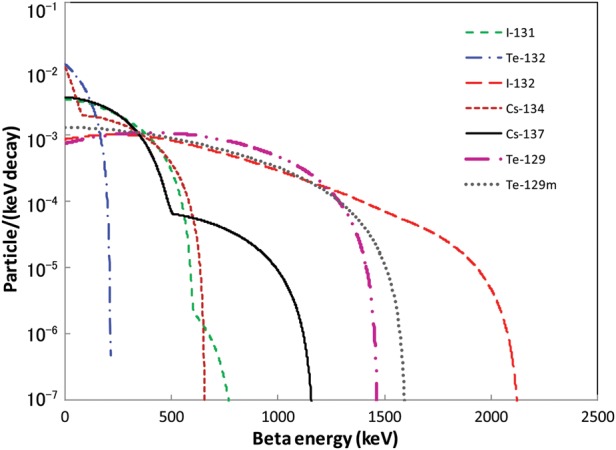
Beta-ray energy spectra for ^129m^Te, ^129^Te, ^131^I, ^132^Te, ^132^I, ^134^Cs and ^137^Cs.

The energy deposition in the air and in ground soil by β-rays from deposited radionuclides was simulated using a numerical method proposed and validated previously [5]. The energy spectra of β-rays emitted from the radionuclides of interest (^129m^Te, ^129^Te, ^131^I, ^132^Te, ^132^I, ^134^Cs and ^137^Cs) were determined from the β-decay phase space [6] (Fig. 1). Strontium-90, which has long half-life (28.6 y), is an important radionuclide for the β-ray exposure of fission products. However, on the FDNPP accident, the deposition density ratio of ^90^Sr/^137^Cs is reported to be 1/100–1/1000 [7], therefore ^90^Sr is not taken into account in this study. The transport of the β-rays was simulated with the Monte Carlo N-Particle transport code version 4C (MCNP-4C) [8]. The simulation technique was otherwise identical to that published previously [2, 3].

### Calculation geometry

**Fig. 2.**
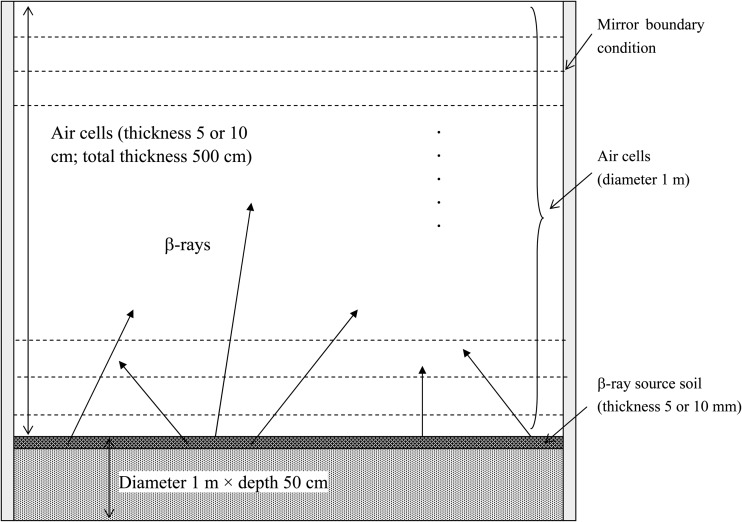
Monte Carlo input geometry.

Computations of radiation dose in the air and in/on the ground by β-rays emitted from radionuclides in the ground soil were based on a cylindrical structure (diameter 1 m) [2, 3], as shown in Fig. 2. The bottom of the cylinder was represented by a soil layer (thickness 50 cm), where radionuclides were distributed uniformly on the surface at a depth of 5 mm and 10 mm. Repeating air layers were modeled above the ground up to 5 m (the first air layer was 5 cm thick, others were 10 cm thick). A mirror boundary condition was used at the lateral wall of the cylinder, shown in Fig. 2, to avoid particle loss and reduce computation time. By using this approach, the cylindrical geometry could be approximated by infinite planes of soil and air. The radiation that escaped from the lateral wall was treated as entering the cylindrical geometry again at the same location on the lateral wall with the same energy and angle as when it escaped. The energy deposition in the air layers at various heights above the ground was computed similarly to obtain the air dose.

### Dose calculation

The β-ray dose at the time of deposition of each radionuclide *D*^*0*^_*i*_(Gy/Bq) is calculated from deposited energy ε_*i*_(*h*) (J) in each air or soil cell, and the energy deposition in the cell is divided by each mass *m*(*h*) (kg) at height *h* and the β-ray emission rate per decay α_*i*_ as follows:

(1) $$\eqalign{D_i^0 (h)=\,{{\varepsilon _i (h)} \over {m(h)}} \cdot \alpha _i \; ( {i=^{{\rm 129m}} {\rm\! Te},^{{\rm 129}} {\rm\! Te},^{{\rm 131}}} {\rm I},  ^{{\rm 132}} {\rm Te},^{{\rm 132}} {\rm\! I},^{{\rm 134}} {\rm\! Cs \ and \ }^{{\rm 137}} {\rm Cs}} ). $$

The β-ray dose rate $\dot D_i^0$ ((μGy/h)/Bq) at the time of deposition of each radionuclide is given by

(2) $$\dot D_i^0 (h)=D_i^0 (h) \cdot r_h \cdot r_c , $$

where *r*_*h*_ is the unit conversion factor for seconds to hours (3600 s/h) and *r*_*c*_ is the unit conversion factor for Gy to μGy (10^6^). Time changes of dose rate $\dot D_s (t,h)$ for each radionuclide are considered in terms of the decay constant λ_*i*_ of each radionuclide. To obtain the β-ray dose at conditions of 1000 kBq/m^2 137^Cs deposition, such that deposition density is similar to that in the region between Iitate Village and Fukushima City, the deposition ratio of radionuclide *i* to ^137^Cs and the decay constant λ_*i*_ are considered:

(3) $$\dot D_s (t,h)=\sum\limits_i {\dot D_i^0 } (h) \cdot f_i \cdot \exp ( - \lambda _i t). $$

The ratio *f*_*i*_ and decay constant λ_*i*_ for each radionuclide, (*i* = ^129m^Te, ^129^Te, ^131^I, ^132^Te, ^132^I, ^134^Cs and ^137^Cs) were taken from the literature [1, 9, 10, 11], and the values used are listed in Table 1.

**Table 1:** Half-life, decay constant, and radioactivity ratio to ^137^Cs for the FDNPP accident [1, 9, 10, 11]

Nuclide	Half-life	Decay constant (s^−1^)	Radioactivity ratio to ^137^Cs	
			Assumed	From data
^129m^Te	33.6 d	2.388 × 10^−7^	1.0	1.0–1.08
^129^Te^a^	33.6 d	2.388 × 10^−7^	0.7	0.68–0.73
^131^I	8.02 d	1.000 × 10^−6^	9.2	7.3–9.2
^132^Te	3.204 d	2.504 × 10^−6^	8.3	5.7–8.3
^132^I^a^	3.204 d	2.504 × 10^−6^	8.3	5.6–8.3
^134^Cs	2.065 y	1.064 × 10^−8^	1.0	0.95–1.03
^137^Cs	30.04 y	6.096 × 10^−7^	1	1

^a^The half-lives are taken to be those of the parent radionuclide.

### Calculation of 70 µm dose

To estimate the skin dose on a small target at the millimeter scale, the depth dose was simulated with the ICRU sphere [diameter 30 cm; density 1 g cm^−3^; elemental composition: H (10.1 wt%), C (11.1 wt%), O (76.2 wt%), N (2.6 wt%)] geometry. The same radionuclides were assumed to exist on a tissue-equivalent plastic sphere (ICRU sphere, diameter 30 cm), after which β-rays were transported [2, 3]. The energy deposition accumulated at each depth, and the attenuation coefficient at a given depth was computed by using the ratio between β-ray dose at the depth of interest and that on the surface. The time-dependent attenuation factor was calculated using the same technique as in Eq. 3.

## RESULTS

### Beta-ray air dose

**Fig. 3.**
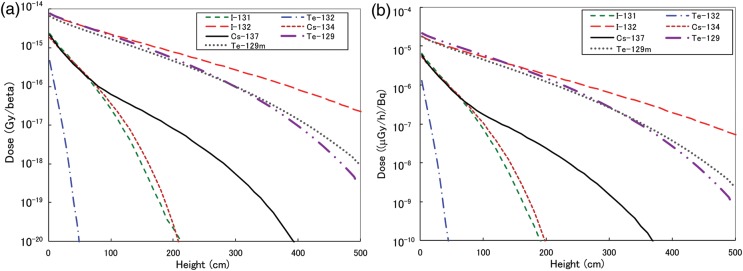
Calculated result at deposition for **(a)** β-ray dose per β emission of ^129m^Te, ^129^Te, ^131^I, ^132^Te, ^132^I, ^134^Cs and ^137^Cs; and **(b)** β-ray dose rate per 1 Bq of ^129m^Te, ^129^Te, ^131^I, ^132^Te, ^132^I, ^134^Cs and ^137^Cs.

The calculation results of the β-ray dose per β emission and the β-ray dose rate (μGy/h) for 1 Bq for each nuclide *D*^*0*^_*i*_ as a function of height in a condition of 5 mm uniform source geometry are shown in Fig. 3a and b, respectively. Iodine-132, ^129m^Te and ^129^Te, the β-rays of which have relatively high maximum energies (Fig. 2), occur in higher doses on the surface and also show height dependence. In contrast, low-energy β-rays from ^132^Te show a rapid decrease with height.

**Fig. 4.**
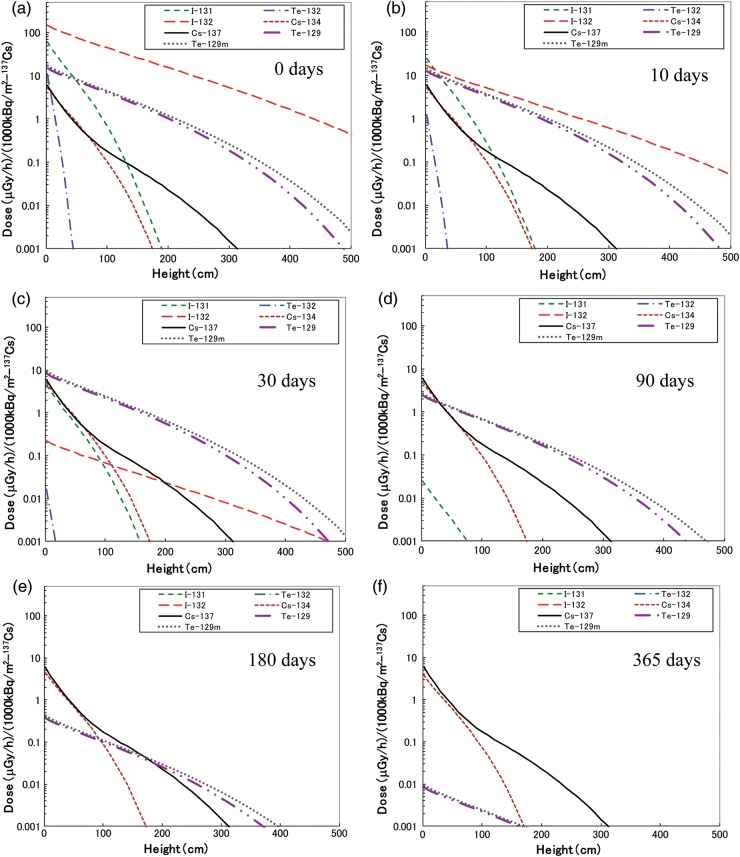
Dose rate change as a function of height from the ground in a condition of 1000 kBq/m^2 137^Cs deposition. **(a–f)** indicate 0, 10, 30, 90, 180 and 365 days after deposition, respectively.

The β-ray dose rate changes with time from deposition. To show the order of dose rate for the FDNPP accident, the β-ray dose rate for 1000 kBq/m^2^ of ^137^Cs deposition with a 5 mm uniform source was calculated according to Eq. 3. This concentration is similar to that in the region between Iitate Village and Fukushima City. The calculation results of the dose rate as a function of height from the ground given 1000 kBq/m^2^ of ^137^Cs deposition are shown in Fig. 4a–f, which correspond to 0, 10, 30, 90, 180 and 365 days after deposition, respectively.

Iodine-132 is the primary contributor to β-ray air dose for several days after deposition, followed by ^131^I. These two radioactive iodine isotopes have short half-lives (3.204 and 8.02 days, respectively) as listed in Table 1. Therefore, about 30 days after deposition the contributions of both ^131^I and ^132^I to the dose rate lowered, at which time ^129m^Te and ^129^Te instead constituted the majority of the air dose. After several months, the dominant radionuclides were ^134^Cs and ^137^Cs due to their long half-lives (2.06 and 30.02 years, respectively).

For a 5 mm uniform source, the dose rate in air at the ground surface at 0, 10, 30, 90, 180 and 365 days after deposition was 240, 81, 33, 16, 11 and 9.7 µGy/h, respectively. This shows that much higher β-dose rates in air than γ rays were estimated by this calculation.

### Beta-ray dose in soil and on the ground surface

**Fig. 5.**
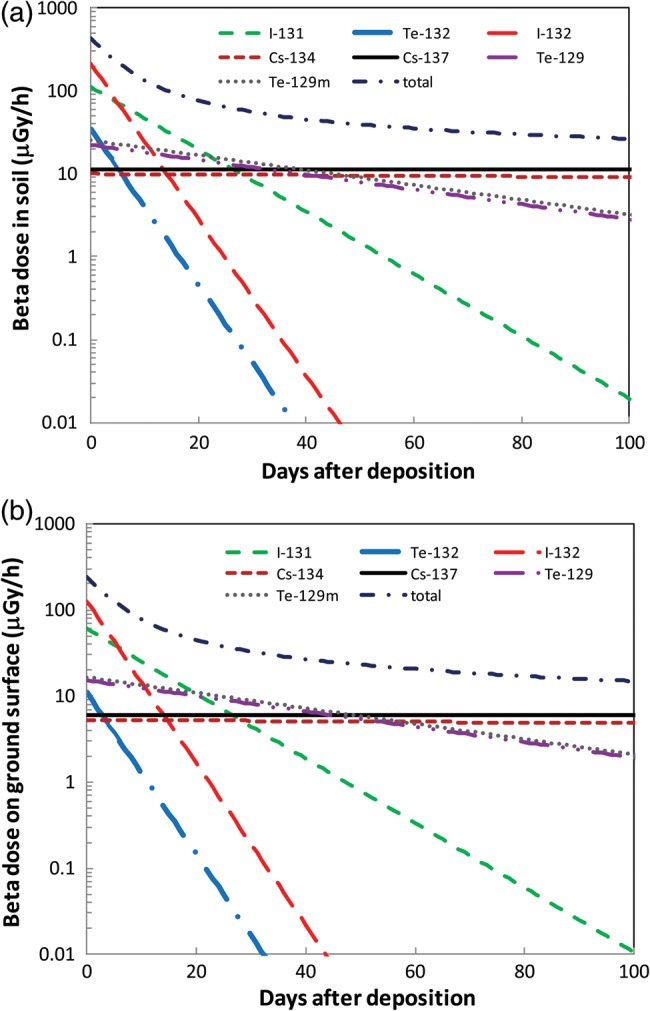
Dose rate change according to the time after deposition in the case of 1000 kBq/m^2 137^Cs deposition **(a)** in soil, and **(b)** at 2.5 cm from the ground surface.

The calculated β-ray dose rates in soil and on the ground surface as a function of time are shown in Fig. 5a and b, respectively. The dose rate in soil at deposition is almost 430 µGy/h, and this fell to ∼ 18 µGy/h after 1 year. The β-ray dose rate in soil was 1.7–1.9-fold higher than that on the ground surface.

### Ratio of β-ray dose to γ-ray dose

**Fig. 6.**
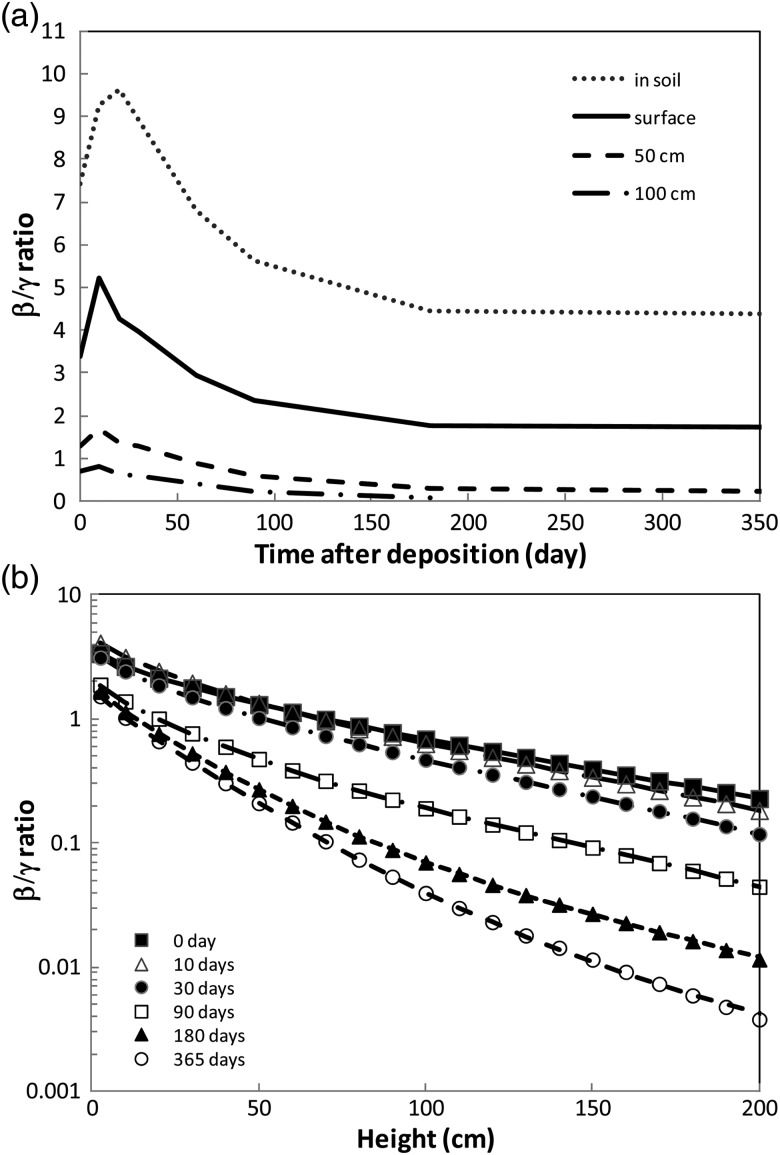
Beta-ray to γ-ray dose rate ratio **(a)** as a function of time after deposition, and **(b)** as a function of height. Curves show the fitting results with χ^2^ less than 1 × 10^−4^.

A β-ray to γ-ray dose ratio is useful for reconstructing the β-ray dose from the γ-ray dose survey monitoring data. The γ-ray dose value is taken from Imanaka *et al.* [9]. The β-ray to γ-ray dose ratio has been obtained for conditions in soil, on the ground surface, and 50 and 100 cm above the ground, as shown in Fig. 6a. The ratio as a function of height is shown in Fig. 6b. The estimated β-ray dose rate is ∼ 10-fold (resp. 5-fold) higher than the γ-ray dose rate in soil (resp. on the ground surface) at ∼ 20 days after deposition, and ∼ 4-fold (resp. 1.7-fold) higher after 6 months or more.

The ratio seen in Fig. 6a has a broad peak at ∼ 20 days, but after 6 months the ratio levels off. For convenience, the height (*h*) dependence of the ratio *r*(*h*) up to 200 cm shown in Fig. 6b was fitted with parameters of *a*, *b*, *c*, *d*, *e* and *f* as follows:

(4) $$r\left( h \right) = a{\rm exp}\left( { - b h} \right) + ch^{ - d} {\rm exp}\left( { - e h} \right) + f. $$

These fitted parameters are listed in Table 2. The fitted curves in Fig. 6b show good reproducibility with the obtained β/γ ratio.

**Table 2:** Fitted parameters

Time (day)^a^	*a*	*b*	*c*	*d*	*e*	*f*
0	1.908	0.0106	1.917	0.1608	0.03427	−0.0014
10	1.864	0.0112	2.796	0.115	0.03639	−0.015
30	1.533	0.0119	2.024	0.1043	0.0384	−0.025
90	0.605	0.0122	1.538	0.0959	0.0389	−0.0085
180	0.189	0.0140	1.771	0.0897	0.0391	0.00012
365	0.127	0.0192	1.687	0.0896	0.0401	0.0012

Fitting χ^2^ was less than 1 × 10^−4^. ^a^Days after deposition.

### Beta-ray air dose ratio of 10 mm to 5 mm uniform source

**Fig. 7.**
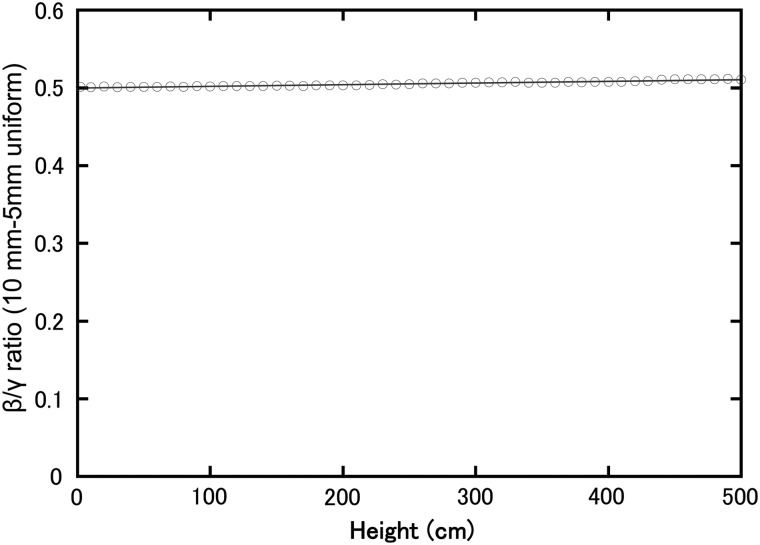
Beta-ray to γ-ray dose rate ratio of 10 to 5 mm uniform source.

Calculation of the dose with a 10 mm uniform source was also performed, and the result was approximately half the value of that calculated with a 5 mm uniform source. The ratio between the results of the 10 and 5 mm uniform source calculations is shown in Fig. 7. The ratio became slightly greater than 0.5 as the height increased, demonstrating that high-energy β-rays penetrate to higher locations. However, as a height of 200 cm is sufficient for the purposes of discussing β-ray dose on insect exoskeleton or human skin, the β-ray dose from the 10 mm uniform source could be estimated by halving the β-ray dose from the 5 mm uniform source.

### Calculation of 70 µm dose

**Fig. 8.**
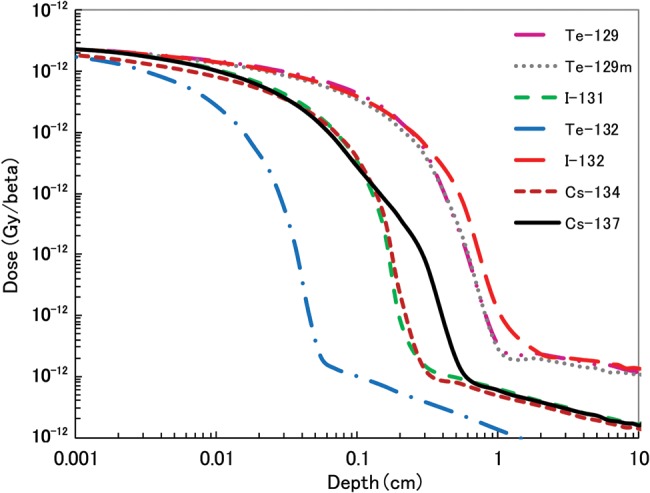
Beta-ray dose per β emission as a function of depth for ^129m^Te, ^129^Te, ^131^I, ^132^Te, ^132^I, ^134^Cs and ^137^Cs.

The results of β-ray dose per β emission as a function of depth for ^129m^Te, ^129^Te, ^131^I, ^132^Te, ^132^I, ^134^Cs and ^137^Cs are shown in Fig. 8. The β-ray dose for each radionuclide is decreased by a factor of 10^−5^ within 1 cm depth, illustrating that the β-ray dose of these radionuclides does not contribute to the effective dose.

**Fig. 9.**
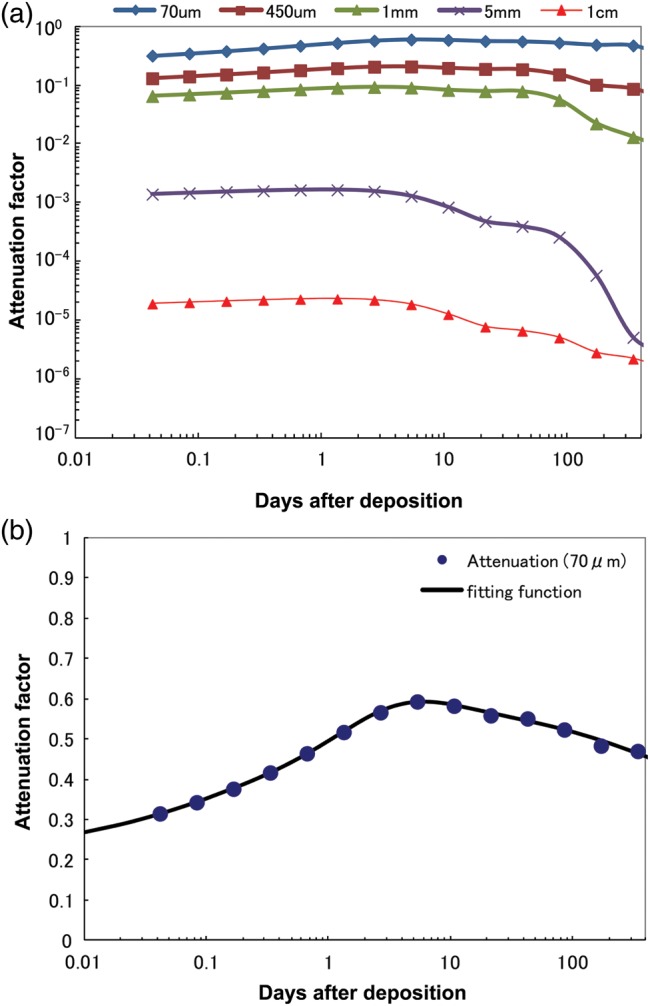
Attenuation factor as a function of time. **(a)** 70 µm, 450 µm, 1 mm, 5 mm and 1 cm dose. **(b)** 70 µm dose and fitting function for the data calculated using Eq. 4.

The attenuation factors as a function of time from 0–400 days after deposition are shown in Fig. 9a and b. Figure 9a shows the attenuation factors for five depths: 70 µm, 450 µm, 1 mm, 5 mm and 1 cm. Figure 9b shows only the 70 µm attenuation factor on a linear scale. For convenience, least-square fitting was performed for the 70 µm attenuation factor with the following equation:

(5) $$\alpha (t)=a_1+a_2 \cdot \exp {\rm \{ }(\log (t ) - a_3 )^2 /a_4 ^2 \}+a_5 \cdot \exp \{ (\log (t) - a_6 )^2 /a_7 ^2 \}. $$

**Fig. 10.**
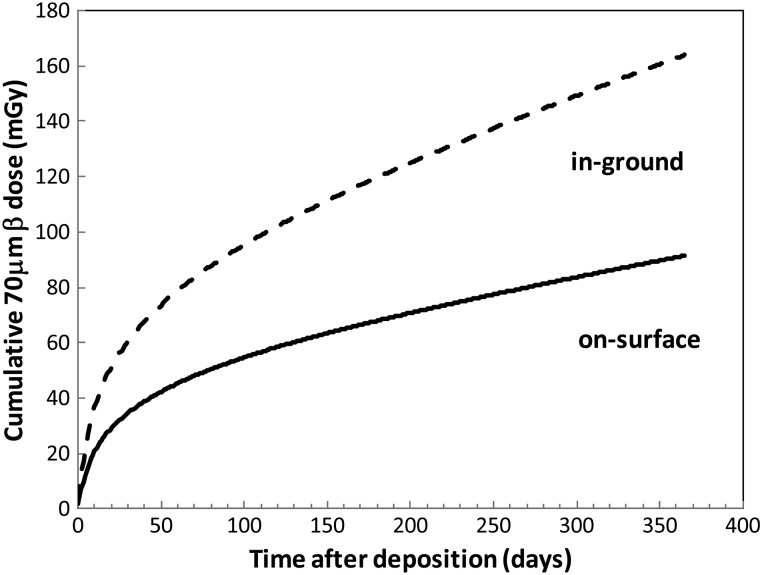
Cumulative 70 µm β-ray dose on the ground surface and in the soil after deposition in a condition of 1000 kBq/m2 137Cs deposition.

The fitted parameters are listed in Table 3. The 70 µm β-ray dose can be estimated by multiplying this attenuation factor and the tissue/air kerma ratio (1.09) by the above-mentioned surface dose. For example, the cumulative 70 µm β-ray dose on the ground surface and in the soil were calculated and shown in Fig. 10. The cumulative 70 µm β-ray dose in soil is about twice that on the ground surface. It shows that insects that burrow into the soil are exposed to a higher β-ray dose. The resultant cumulative 70 µm β-ray doses at 30, 60 and 90 days after deposition are estimated to be 35, 45 and 53 mGy on the ground surface and 61, 79 and 92 mGy in soil, respectively.

**Table 3:** Results of least-square fitting of the 70 µm attenuation factor with Eq. 4

Parameter	Value	Error
*a*_*1*_	0.220	0.0392
*a*_*2*_	0.0609	0.0119
*a*_*3*_	0.5847	0.0622
*a*_*4*_	0.6424	0.1197
*a*_*5*_	0.3281	0.0390
*a*_*6*_	1.246	0.0586
*a*_*7*_	2.359	0.2461
χ^2^	0.00028

## DISCUSSION

In the case of γ rays, ^132^I, ^131^I, ^134^Cs and ^137^Cs are the main contributors to the γ-dose rate in air, depending on the time after deposition [1, 9]. In contrast, ^129m^Te and ^129^Te also contribute to the β-ray dose rate in the early stages after deposition has occurred. The half-lives of ^129m^Te and ^129^Te (33.6 d) are slightly longer than those of ^132^I and^131^I (3.204 d and 8.02 d). When we discuss the β-ray exposure from the deposited radionuclides of the FDNPP accident, ^129m^Te and ^129^Te cannot be neglected. On the other hand, ^90^Sr, which has long half-life (28.6 y), is an important radionuclide for the β-ray exposure of fission products. In the case of radionuclides uniformly distributed over a 100 cm^2^ circular area, the skin dose conversion factor for ^90^Sr and ^90^Y (1.478 and 1.775 (μGy·h^−1^·Bq^−1^·cm^2^)) is roughly the same as that for ^137^Cs (1.537 (μGy·h^−1^·Bq^−1^·cm^2^)) [12]. For the FDNPP accident, the deposition density ratio of ^90^Sr/^137^Cs is reported to be 1/100–1/1000 [7] so the skin dose due to ^90^Sr-^90^Y is expected to be 0.1–1% of the skin dose due to ^137^Cs. Furthermore, the half-life of ^90^Sr (28.6y) is shorter than that of ^137^Cs (30.04y). Therefore, the ^90^Sr β-ray contribution is negligible in the early stages after the FDNPP accident.

In this study, the ratio of the β- to γ-ray dose rate in air was obtained using an empirical fitting function, as indicated in Table 2. This result was quite useful for estimating the β-ray dose in air from the γ-ray dose, because a lot of monitoring data has been accumulated for the γ-ray dose in air [1, 9, 10, 13]. However, an assumption about the radioactivity ratios has been used in this study (see Table 2); this assumption can be used for a relatively wide area, except for part of Minami-Soma and Iwaki Cities [1, 11].

The calculation example of a 70 µm dose (Fig. 10) corresponds to the skin dose for barefoot walking on 1000 kBq/m^2 137^Cs-contaminated ground. The resultant cumulative 70 µm β-ray doses at 30–90 days after deposition are estimated to be 35–53 mGy on the ground surface and 61–92 mGy in the soil, respectively. While this cumulative β-ray dose is not a realistic measure of human exposure, as humans do not typically come into barefoot contact with the ground throughout the day, it is realistic for insects, which often stay in or on the ground.

## CONCLUSION

This paper has shown the method and results of our β-ray dose calculation for the dosimetry of the FDNPP accident. In summary, we calculated the β-ray dose in air and in soil for a deposition density of 1000 kBq/m^2 137^Cs after the FDNPP accident, using an MCNP simulation. The ^129m^Te, ^129^Te, ^131^I, ^132^Te, ^132^I, ^134^Cs and ^137^Cs deposition densities were assumed to be in a ratio of 1:0.7:9.2:8.3:8.3:1:1. The β-ray dose rate was ∼ 10-fold (resp. 5-fold) higher than the γ-ray dose rate in soil (resp. on the ground surface) at ∼ 20 days after deposition, and ∼ 4-fold (resp. 1.7-fold) higher after 6 months or more. For convenience, the height dependences of the ratios for 0, 10, 30, 90, 180 and 365 days were obtained by a fitting function, as was the 70 µm attenuation factor. These results can be used for β-ray dose estimation, not only for small creatures such as insects but also for human skin. The cumulative 70 µm β-ray doses at 30, 60 and 90 days after deposition were estimated to be 35, 45 and 53 mGy on the ground surface, and 61, 79 and 92 mGy in the ground soil, respectively.

## FUNDING

Funding to pay the Open Access publication charges for this article was provided by Hiroshima University.
